# Prospective Clinical Trial for Septic Arthritis: Cartilage Degradation and Inflammation Are Associated with Upregulation of Cartilage Metabolites

**DOI:** 10.1155/2016/5491971

**Published:** 2016-09-05

**Authors:** Hagen Schmal, Anke Bernstein, Matthias J. Feucht, Benjamin Erdle, Jan M. Pestka, That Minh Pham, Eva Johanna Kubosch

**Affiliations:** ^1^Department of Orthopaedics and Traumatology, Odense University Hospital, Odense, Denmark; ^2^Department of Clinical Research, University of Southern Denmark, Odense, Denmark; ^3^Department of Orthopedics and Trauma Surgery, Albert-Ludwigs University Medical Center Freiburg, Freiburg, Germany

## Abstract

*Background*. Intra-articular infections can rapidly lead to osteoarthritic degradation. The aim of this clinical biomarker analysis was to investigate the influence of inflammation on cartilage destruction and metabolism.* Methods*. Patients with acute joint infections were enrolled in a prospective clinical trial and the cytokine composition of effusions (*n* = 76) was analyzed. Characteristics of epidemiology and disease severity were correlated with levels of cytokines with known roles in cartilage turnover and degradation.* Results*. Higher synovial IL-1*β* concentrations were associated with clinical parameters indicating a higher disease severity (*p* < 0.03) excluding the incidence of sepsis. Additionally, intra-articular IL-1*β* levels correlated with inflammatory serum parameters as leucocyte counts (LC) and C-reactive protein concentrations (*p* < 0.05) but not with age or comorbidity. Both higher LC and synovial IL-1*β* levels were associated with increased intra-articular collagen type II cleavage products (C2C) indicating cartilage degradation. Joints with preinfectious lesions had higher C2C levels. Intra-articular inflammation led to increased concentrations of typical cartilage metabolites as bFGF, BMP-2, and BMP-7. Infections with* Staphylococcus* species induced higher IL-1*β* expression but less cartilage destruction than other bacteria.* Conclusion*. Articular infections have bacteria-specific implications on cartilage metabolism. Collagen type II cleavage products reliably mark destruction, which is associated with upregulation of typical cartilage turnover cytokines. This trial is registered with DRKS00003536, MISSinG.

## 1. Introduction

Intra-articular infections are deleterious for near-term joint function and cause pain. Furthermore, they can lead to long-term osteoarthritic degradation, suggesting an association of acute inflammation and a catabolic cartilage metabolism. The regulatory mechanisms behind this are not yet fully understood. Until now, most data available is about the association of chronic inflammatory diseases as rheumatoid arthritis and cartilage degradation, describing causal roles for proinflammatory cytokines such as TNF*α*, IL-6, or death receptor 3 [1]. Furthermore, direct toxic effects of bacterial toxins on chondrocytes causing their death have been described [2]. Moreover, toll-like receptors (TLR) do exhibit both proinflammatory and catabolic effects mediated by the NF-*κ*B pathway in septic arthritis, an example for overlapping functions in regulatory pathways [3]. The interactions between mediators upregulated during infection and cartilage metabolism need to be clarified in order to develop effective interventional strategies. Therefore, the aim of this clinical trial was to correlate parameters defining the severity of inflammation with proteins characterizing cartilage degradation and metabolism. We hypothesized that there is a statistically significant association between certain clinical symptoms, synovial expression of inflammatory mediators, cartilage degradation, and cytokines with known importance in cartilage metabolism.

## 2. Material and Methods

### 2.1. Clinical Trial

The trial was registered (MISSinG, DRKS00003536) and approved by the Ethics Board of the University of Freiburg (AN-EK-FRBRG-50/11). All patients participating in this study provided their written consent.

As already described in previous publications [4, 5], a consecutive series of 75 patients treated between April 2011 and November 2012, presenting the clinical symptoms of bacterial joint infection, were recruited for the prospective collection of joint fluid. All patients suffered from pain, swelling, effusion, and elevated inflammatory serological parameters (e.g., C-reactive protein). Effusions from 76 affected joints were included in the analysis. Infections of knee (75%), hip (6.6%), ankle (1.3%), and shoulder (17.1%) were included. Four other patients had to be excluded because of storage-protocol violations (*n* = 3) or sudden death because of fulminant lung embolism (*n* = 1, no written consent).

### 2.2. Sample Storage

Effusions were obtained within the first 24 hours after diagnosis during arthroscopy or preoperative puncture and immediately frozen. Specimens were stored in liquid nitrogen until analysis.

### 2.3. Analyzed Parameters

Age (time point of index diagnosis and puncture), sex (male/female), body mass index (BMI), and smoking habits (yes/no) were the epidemiological parameters characterizing the patients. For description of the septic constellation the following serum values were recorded: initial (time point of diagnosis and puncture), maximal and final (time point demission) leucocyte counts, and C-reactive protein (CRP). Furthermore, the degree of systemic inflammation was evaluated using the following parameters: necessity for an intensive care treatment, documented diagnosis of sepsis (fulfilment of clinical sepsis criteria), fulfilment of clinical empyema criteria (synovitis ≥ grad 2 [6], detection of intra-articular bacteria, and clinical necessity of recurrent lavage), and necessity of in-hospital treatment. The comorbidity was assessed using the ASA classification (physical status according to the American Society of Anesthesiologists). Furthermore, the preinfectious joint damage was recorded, which was defined as any articular lesion including osteoarthritis. The evaluation of the Kellgren Lawrence Score [7] was based on conventional X-rays and done by 3 independent orthopedic surgeons, resulting in a consensus decision. 81% of isolated bacteria belonged to* Staphylococcus* species; in the subgroup of patients with knee infections, the portion of isolated* Staphylococcus* species increased to 86%.

### 2.4. ELISA and Protein Content

Cytokine and protein concentrations (interleukin- [IL-] 1*β*, IL-10, aggrecan [ACAN], basic fibroblast growth factor [bFGF], bone morphogenetic protein- [BMP-] 2 and BMP-7, and collagen type 2 cleavage [C2C]) in synovial joint fluids from acutely infected knee joints or coculture supernatants were analyzed by ELISA (RnD, Minneapolis, MN, USA, and BioSource Deutschland GmbH, Solingen, Germany) according to the manufacturers' instructions. The Nitrotyrosine ELISA Kit was purchased from Abcam (Cambridge, UK). Briefly, this assay employs the quantitative sandwich enzyme immunoassay technique. The microplate was precoated with a specific monoclonal antibody. Supernatants were applied to the wells and, after washing, an HRP-conjugated specific antibody was added to the wells. Following the next wash, color development was proportional to the protein concentration and calculated by comparison with a standard. A colorimetric method was applied to quantify total protein amount in the lavage fluids. The bicinchoninic acid (BCA) assay was available in kit form from Pierce (Rockford, IL, USA) and used according to the manufacturer's instructions [4]. All data from the analyzed cytokines and proteins are reported as relative expression to the total protein content. Statistical calculations were based on these values.

### 2.5. Data Analysis and Statistics

Concentrations of proteins and cytokines determined by the specific ELISAs and the BCA assay were calculated according to the manufacturers' instructions (RnD, Minneapolis, MN, USA; Thermo Scientific, Rockford, IL, USA), creating a standard curve and reducing data using a four-parameter logistic (4-PL) curve fit by using GraphPad Prism 5 software (GraphPad Software, Inc., La Jolla, CA, USA). All values were expressed as mean ± standard error of the mean. Regarding the scores and all numerical values, statistical significance was tested nonparametrically primarily using the *U*-test according to Mann and Whitney. Multiple comparisons were calculated using a post hoc statistics based on the *H*-test according to Kruskall-Wallis. Correlations were determined by calculating the Spearman coefficient (*ρ*) for the predominantly not normally distributed values. A cluster analysis with a simple agglomeration method was used for grouping values of registered cases on the basis of minimal distances between group members. This in combination with a percentile analysis or known boarders of values (leucocyte counts and CRP) was used to group values. Incidences were compared using the chi square test. Statistical significance was defined as *p* < 0.05.

## 3. Results

### 3.1. Characterization of Included Patients and Grouping

75 patients with 76 articular infections were included in a prospective clinical trial. The average age was 59.7 ± 2.5 years, the average BMI was 27.8 ± 0.7, and the ratio of female and male patients was 67.1%/32.9%. The distribution of the ASA classification, characterizing the patient's comorbidity, was as follows: group 1: 26.3%, group 2: 27.6%, group 3: 42.1%, and group 4: 3.9%. In order to gain a more homogenous population, patients with exclusive knee infections were separately analyzed (*n* = 57). The average age of this group was 57.2 ± 3.1 years, the average BMI was 27.5 ± 0.9, and the ratio of female and male patients was 73.7%/26.3% (no statistically significant difference between the analysis groups for each item). The distribution of the ASA classification, characterizing the patient's comorbidity, was as follows: group 1: 29.8%, group 2: 28.1%, group 3: 38.6%, and group 4: 3.5%. All included patients survived, and all infections were successfully treated; two times an arthrodesis was necessary.

### 3.2. Synovial IL-1*β* Levels in relation to Clinical Parameters Characterizing the Severity of Infection

Based on a cluster analysis, the intra-articular IL-1*β* levels were regarded as increased when the relative expression was higher than 0.005. This correlated with the median. The necessity of intensive care or an in-hospital treatment was associated with increased synovial IL-1*β* levels in all patients and in the subgroup of septic knee arthritis. In contrast, in case of a clinically documented sepsis, intra-articular IL-1*β* concentrations were not elevated. If the patients fulfilled the clinical empyema criteria, the majority had synovial IL-1*β* levels above the median. Furthermore, a preinfectious joint damage, which was defined as any lesion including osteoarthritis, predisposed to higher intra-articular IL-1*β* concentrations indicating a higher degree of inflammation. Data is summarized in Table 1. The comorbidity did not influence the degree of inflammatory response; there was no correlation of any CRP level or the synovial IL-1*β* concentrations with the ASA classification, and the comorbidity was equally distributed in the IL-1*β* clusters in both all and knee infections.

### 3.3. Association of Relative Intra-Articular IL-1*β* Levels with Systemic Inflammatory Parameters

To describe the association of joint inflammation and the systemic inflammatory reaction, serum and blood levels of C-reactive protein (CRP) and leucocyte counts were correlated with synovial IL-1*β* levels. The course of septic arthritis was characterized using the initial, the maximal, and the final (discharge) systemic values. There was no association with the assessments at the time point of discharge, which was expected, because the inflammation was successfully treated. However, both initial and maximal CRP serum concentrations and leucocyte counts correlated with intra-articular IL-1*β* concentrations. Data is summarized in Table 2. Nitrotyrosine (NO-Tyr) is a known marker of inflammation and NO production, which is also known to be associated with cartilage destruction [8]. Therefore, we have included an analysis of this marker in the study. NO-Tyr failed to show any correlation to clinical, serological, or other secretory inflammatory markers.

### 3.4. Characterizing Cartilage Degradation in Septic Arthritis

**Figure 1:**
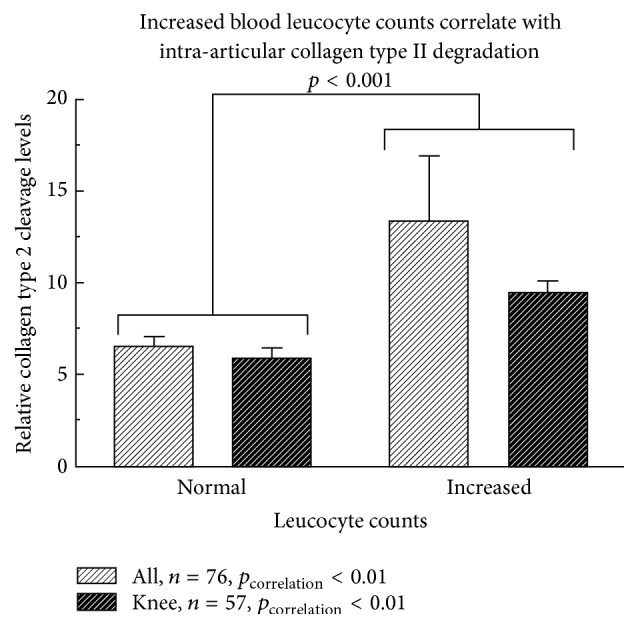
Increased serum leucocytes at time point of diagnosis were associated with higher collagen type 2 cleavage levels (C2C), a cartilage breakdown product. This reached statistical significance analyzing all patients and the subgroup of knee infections. Reported are the C2C levels relative to the total protein content.

**Figure 2:**
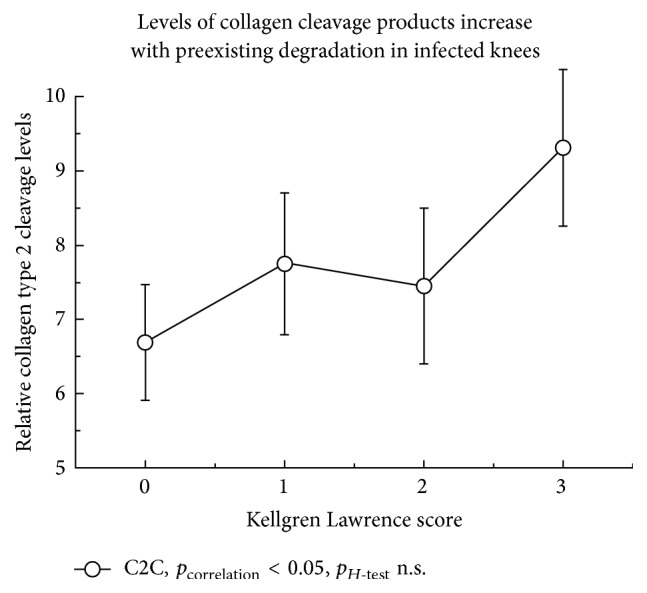
An increasing Kellgren Lawrence Score (KLS), defining progress of osteoarthritis, was associated with enhanced collagen type 2 cleavage levels (C2C), defining cartilage degradation, in septic knee arthritis (*p* = 0.049). The group KLS 3 includes one case with a KLS = 4. Reported are the C2C levels relative to the total protein content.

Degradation of cartilage is characterized by the release of extracellular matrix products as collagen (collagen type 2 cleavage—C2C) or aggrecan. There was a statistically significant correlation of synovial aggrecan (*ρ* = 0.30, *p* = 0.006) and C2C levels (*ρ* = 0.34, *p* = 0.002) with initial serum leucocyte counts in all patients. In the subgroup of knee infections only correlations with C2C concentrations reached statistical significance (*ρ* = 0.45, *p* = 0.0005), which could also be shown for the leucocyte count maximum (*ρ* = 0.41, *p* = 0.002). Increased serum leucocytes at the time point of diagnosis were also associated with higher C2C-levels. This association reached statistical significance analyzing all patients and the subgroup of knee infections. Reported are the C2C levels relative to the total protein content (Figure 1). There was also a positive correlation of C2C concentrations with intra-articular IL-1*β* levels (*ρ* = 0.34, *p* = 0.001), which also characterize synovial inflammation. This could be confirmed in the subgroup of knee infections. An increasing Kellgren Lawrence Score (KLS), which radiologically defines the progress of osteoarthritis (OA), was associated with enhanced C2C levels in septic knee arthritis (*p* = 0.049, Figure 2). The distribution of KLS was 18.0% grade 0, 34.4% grade 1, 27.9% grade 2, 18.0% grade 3, and 1.6% grade 4. OA was considered as a preinfectious joint damage, which was associated with a higher degree of inflammation (synovial IL-1*β* concentrations). Although the regulation pattern was similar for aggrecan, the values failed to reach statistical significance.

### 3.5. Correlation of Proinflammatory IL-1*β* with the Mediators of Cartilage Metabolism bFGF, CD105, BMP-2, and BMP-7 and the Anti-Inflammatory Marker IL-10

**Figure 3:**
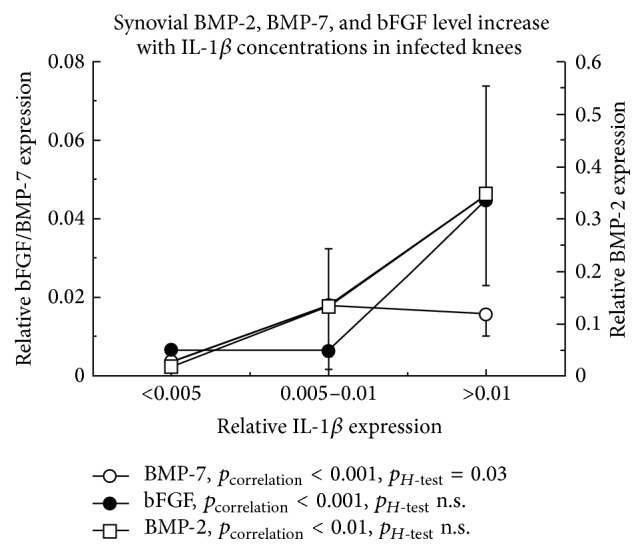
Increased synovial IL-1*β* levels were associated with enhanced bFGF, BMP-2, and BMP-7 concentrations in septic knee arthritis (*p* < 0.001). All protein levels were analyzed relative to the synovial total protein content.

Since intra-articular concentrations of IL-1*β* characterized the clinical relevance and severity of septic arthritis, the correlations to cytokines with known roles in chondrocyte anabolism or catabolism were analyzed, searching for the interaction between inflammation and cartilage metabolism. Whereas the synovial expressions of bFGF (*ρ* = 0.42, *p* < 0.001), BMP-2 (*ρ* = 0.37, *p* = 0.002), and BMP-7 (*ρ* = 0.49, *p* < 0.0001) were positively associated with IL-1*β*, no correlation could be shown for CD105 (*ρ* = 0.16, n.s.). This was found in all patients and the subgroup of septic knee arthritis. The same association was further calculated based on the different clusters of IL-1*β* (Figure 3), showing increasing concentrations of bFGF, BMP-2, and BMP-7 with rising IL-1*β* levels. The *H*-test, defining statistical significance for the cluster differences, was statistically significant only in case of BMP-7 (*p* = 0.03), therefore demonstrating the strongest dependency on proinflammatory conditions. There was no statistically significant correlation of IL-1*β* and IL-10 (*ρ* < 0.01, n.s.).

### 3.6. Influence of Bacterial Strain

**Figure 4:**
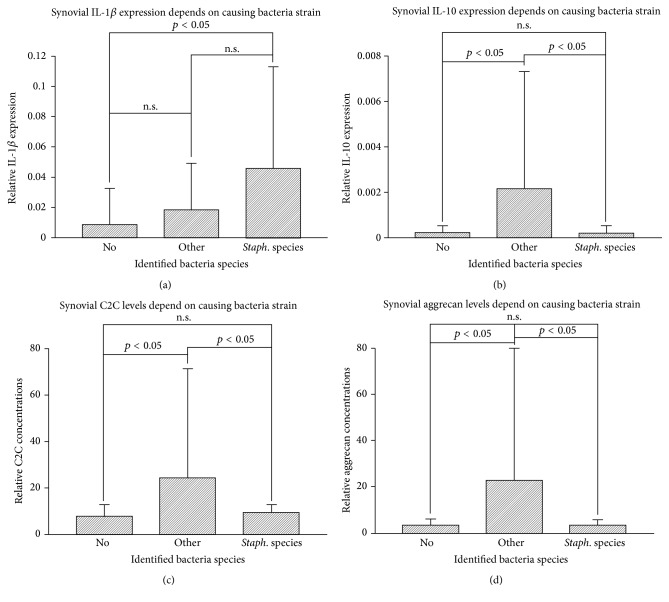
(a) The highest synovial IL-1*β* levels were observed following infections with* Staphylococcus* species. (b) A different expression pattern was observed for IL-10, showing the highest levels in the group following infections with other bacteria. (c) The expression pattern for C2C, indicating cartilage destruction, is similar to IL-10, showing the highest levels in the group following infections with other bacteria. Results represent values × 10^−6^. (d) The expression pattern for another cartilage breakdown marker, aggrecan, is similar to C2C, showing the highest levels in the group following infections with other bacteria. The significance levels indicated represent the ANOVA. no: no detection of bacteria; other: infections with other bacteria;* Staph*. species: infections with* Staphylococcus* species.

In order to analyze the influence of the bacteria species on the inflammatory reaction and cartilage destruction 3 groups were defined: no detection of bacteria (group 1, *n* = 44),* Staphylococcus* species (group 2, *n* = 23), and other bacteria species (group 3, *n* = 9). Infections with* Staphylococcus* species induced the highest expression of IL-1*β* (Figure 4(a)). An ANOVA was used as a screening test, showing statistically significant differences only between group 1 (no) and group 2 (*Staph*. species), which was confirmed by the direct comparison (*p* < 0.001). The anti-inflammatory IL-10 showed a different regulatory pattern with highest levels in the group with other bacteria (Figure 4(b)). Although the ANOVA showed statistically significant differences between group 3 and both other groups, the direct comparison could only confirm this for groups 1 and 3 (*p* = 0.02). C2C and aggrecan, both indicating cartilage breakdown, demonstrated the same associations as IL-10, showing the highest concentration after infections with other bacterial strains (group 3). Although the ANOVA showed again statistically significant differences between group 3 and both other groups, the direct comparison could only confirm this for groups 1 and 3 (*p* = 0.02 and 0.024, resp.). There were no differences for the expression of CD105, bFGF, BMP-2, and BMP-7 between the groups.

## 4. Discussion

The study's main findings are that synovial IL-1*β* levels appear to be a reliable tool to measure the severity and clinical relevance of septic arthritis. Collagen type II cleavage products (C2C) characterize cartilage degradation during acute joint infection better than other matrix breakdown products. Moreover, there is a positive correlation of the degree of intra-articular inflammation with upregulation of bFGF, BMP-2, and BMP-7, representing markers of cartilage metabolism. Regulation of inflammation and cartilage destruction are specifically associated with certain types of bacteria.

Bacterial septic arthritis is frequently found in patients with a high comorbidity, can lead to joint destruction, and is associated with a high mortality. Synovial concentrations of the proinflammatory markers TNF*α*, IL-1*β*, and IL-6 are elevated in the course of disease, in which TNF*α* was a better indicator to discriminate bacterial arthritis from other inflammatory arthritis [9]. The clinical relevance of intra-articular IL-1*β* levels could be confirmed by our data, showing an association with systemic inflammatory parameters as serum leucocyte counts and serum CRP-levels. This has been demonstrated before in the postoperative follow-up of patients undergoing surgical cartilage regenerating procedures [10]. Furthermore, elevated synovial IL-1*β* levels were more frequently found in patients with necessity of intensive care or in-hospital treatment and clinical empyema signs. In contrast, this could not be shown for patients with clinical sepsis signs. This indicates that sepsis in general seems to be associated with physical conditions beyond joint infection. Comorbidity, for example, associated with drug abuse or immunosuppression, which was not decisive in the course of joint infection, seems to play a more important role in these patients [11].

The phenomena of synovial inflammation and cartilage destruction occur within the same time frame as shown by the parallel upregulation of inflammatory cytokines and breakdown products of the extracellular matrix. This was found in different studies investigating osteoarthritis [12], psoriatic arthritis [13], and trauma [14] in humans, and different other conditions including septic arthritis in animals [15]. Our data indicate that collagen type 2 cleavage products (C2C), which possess pharmacodynamic and physiological relevance for cartilage degradation [16], are a more reliable parameter characterizing cartilage degradation in septic arthritis compared to aggrecan. However, the levels of this matrix component were closely related to the development of osteoarthritis without acute infection [17]. The interactions between bacterial infection, upregulation of IL-1*β* and IL-6, chondrocyte apoptosis, and cartilage degradation have been earlier described in vitro; however, the analysis of the molecular basis of the interaction of proinflammatory parameters, matrix degradation, and cartilage metabolism in a human clinical trial was missing. On the other hand, the potential benefits of IL-1 inhibition applying IL-1 receptor-antagonist have been highlighted in a clinical trial attenuating the posttraumatic inflammation [18]. Furthermore, there are several studies investigating the possible beneficial effect for corticosteroids in septic arthritis showing promising data, but—similar to what is seen for specific inhibition of inflammatory mediators—current studies do not provide evidence of higher level for treatment efficacy [19]. The hypothesis that there is a relation of acute bacterial inflammation and cartilage degradation as well as cartilage metabolism could now be confirmed presenting this biomarker analysis and may explain the deleterious long-term effects of acute inflammation on the joint integrity. Previously, upregulation of both proinflammatory cytokines and cartilage metabolites has been shown in vivo following LPS-stimulation of mononuclear cells [4]. In this study not only inflammatory cells but also chondrocytes and synoviocytes have actively contributed to the development of inflammation. In fact, the prolonged effects of synoviocyte activation have been hallmarked as a reason for prolonged devastating cartilage effects after elimination of bacteria in other studies [20]. Our data indicate that joints with a preexisting intra-articular damage are more susceptible to inflammation. This is in line with results showing an anti-inflammatory effect of cartilage itself leading to FAS ligand mediated protection of synovial fibroblasts [5]. Therefore, cartilage is not only target but also an attenuating effector in synovial inflammation. One advantage of in vitro studies is that they can describe cell-specific and time-dependent regulation patterns, which indicate early upregulation of IL-1*β* and a delayed increase of BMP-7 levels. Therefore, these cytokines represent initiation of inflammation and a secondary, postponed start of a regenerative phase. The results of our study support the idea that cytokine regulation during septic arthritis is specific for different strains of bacteria. This has been shown, for example, in* Corynebacterium ulcerans* infections [21]. These bacteria have a nonarthritogenic strain (BR-AD22). With regard to statistical necessities, the infectious bacteria in this trial were summarized in groups, showing that inflammation caused by* Staphylococcus* species is associated with a high IL-1*β* expression, but less cartilage destruction. The mechanisms behind this effect cannot be clarified based on this observational study.

Although the time of analysis was defined and prospectively correlated with clinical signs of inflammation, we only present values of a single sample without time courses. This cross-sectional character of the presented analysis certainly limits the interpretation of the results. Furthermore, it is an observational study, which does not allow drawing conclusions with regard to biochemical or physiological interactions. Furthermore, typical limitations of a clinical study have to be taken into account. This includes the limited number and a high diversity of the examined patient population. The ASA classification is a reliable instrument to classify the health status but does not evaluate different metabolic elimination characteristics, for example, seen in kidney or liver failure, which possibly also exhibits an influence on the concentrations of the measured cytokines.

In conclusion, articular infection and synovial inflammation are bacteria-specific and have direct influence on cartilage metabolism. Collagen type II cleavage products reliably mark destruction, which is associated with upregulation of typical cartilage turnover cytokines as bFGF, BMP-2, and BMP-7.

## Figures and Tables

**Table 1:** The overview shows the relative expression of IL-1*β* in relation to clinical parameters. ICU: necessity of intensive care treatment; sepsis: fulfilment of clinical sepsis criteria; empyema: fulfilment of clinical empyema criteria; preinfectious joint damage: any lesion including osteoarthritis; in-hospital treatment: the difference to an outpatient treatment (< or ≥0.005, *χ* ^2^-test; ICU: intensive care unit, n.s.: not significant).

Symptom	Group	*N* (yes/no)	High IL-1*β*—yes (%)	High IL-1*β*—no (%)	*p*
ICU	All	22/54	59.1	29.6	0.008
	Knees	16/41	56.3	29.3	0.029

Sepsis	All	12/64	58.3	34.4	n.s.
	Knees	8/49	50.0	34.7	n.s.

Empyema	All	47/29	51.1	17.2	0.0016
	Knees	34/23	47.1	21.7	0.025

Preinfectious joint damage	All	50/26	48.0	19.2	0.007
	Knees	37/20	43.2	25.0	n.s.

In-hospital treatment	All	69/7	42.0	0.0	0.014
	Knees	51/6	41.2	0.0	0.024

**Table 2:** Association of relative intra-articular IL-1*β* levels with systemic inflammatory parameters (n.s.: not significant).

		*n*	Spearman *ρ*	*p*
Initial leucocyte count	All	71	0.29	0.007
	Knees	54	0.26	0.031

Initial CRP	All	71	0.29	0.007
	Knees	54	0.23	0.049

Maximal leucocyte count	All	61	0.28	0.014
	Knees	47	—	n.s.

Maximal CRP	All	61	0.30	0.010
	Knees	47	—	n.s.

Discharge leucocyte count	All	61	—	n.s.
	Knees	47	—	n.s.

Discharge CRP	All	61	—	n.s.
	Knees	47	—	n.s.
